# Facilitating Community Participants’ Research Engagement: Community Members’ Perceptions of Community-based Research

**DOI:** 10.15344/2394-4978/2015/142

**Published:** 2015-09-23

**Authors:** Alice M. Tse, Donna-Marie Palakiko, Ephrosine Daniggelis, Emily Makahi

**Affiliations:** School of Nursing and Dental Hygiene, University of Hawai‘i at Mānoa, 2528 McCarthy Mall, Honolulu, HI 96825 USA

**Keywords:** Academic-Community Partnership, Academic Partner, Community Expectations, Community Partner, Community-Based Participatory Research

## Abstract

**Objectives:**

To describe the perspectives of community participants about engaging in community-based participatory research, and then to use the information to develop a model to depict the community participants’ perceptions of interfacing with academic researchers.

**Method:**

A diverse group of Native Hawaiian community-dwelling participants engaged in open-ended and semi-structured focus group interviews, addressing community members’ perceptions of community-based participatory research.

**Results:**

Three key areas were identified: (1) reciprocal trustable is needed; (2) perceptions about the purpose, research intent and expectations; (3) expectations of roles and responsibilities of the researcher(s). A model showing the reciprocity between the academic partner and the community partner is needed to establish the full CBPR process is proposed.

**Conclusion:**

The three themes implied the community participants’ expectations of reciprocal relationships. The dimensions influencing community members’ perceptions of community-based research need to be taken into account when academic researchers interface with community participants. Successful community-based participatory research approaches for addressing the challenges of translating research findings into community actions is enhanced when the expectations of community members are taken into account.

## BACKGROUND/INTRODUCTION

The timely transfer of research findings into practice requires the active participation of individuals who are most likely to benefit from such research. For example, in 2004, translational research became a priority in the United States [1]. The National Institutes of Health (NIH) developed the NIH Roadmap for Medical Research. This Roadmap was designed to speed the application of research findings and scientific discoveries into information that is useful to every-day individuals. The ultimate goal of translational research is to improve health outcomes and reduce disparities by engaging members of community groups to translate evidence-based health interventions into health improvement activities that are meaningful and realistic for their group to implement [2].

### What is a Community Group?

A community group is a social unit of any size that shares common values. Embodied or face-to-face communities are usually small. The identity of the participants and their degree of cohesiveness is affected by conditions such as intent, belief, resources, preferences, needs, risks may be present and shared in common.

When members of communities work alongside the researcher to plan and implement the research findings, the activities then make sense for those directly affected, the community members themselves. This strategy assures the empowerment of the community group and the development of ownership of actions to reduce health disparities outcomes.

### Academic-Community Relationships

While the partnering of academic researchers with community groups has shown promising and sustainable results, the continued distrust of researchers and the research enterprise by many racial/ethnic minorities and indigenous populations have been well documented [3,4]. Ethnic and racial populations have experienced generational cultural trauma and racial/ethnic exploitation. Often social research findings portends a negative context and an escalation of mistrust [5]. Yet, ethnic and minority populations are most at risk for poor health outcomes and their participation is vital for translational research studies.

### Community-Based Participatory Research

The CPBR methodology is widely recognized for engaging communities in research [6]. Community-based Participatory Research (CBPR) is a methodology with potential to address challenges of translating research findings into community actions and overcoming the fears of partnering with academic researchers in general. The CBPR method addresses many of the barriers in the traditional researcher-subject relationship, including mistrust [7], challenges to power sharing [8], lack of engagement by the participants, and lack of sustainability of programming in community settings [9].

The practice of CBPR has evolved over the past 20 years as a research methodology that bridges the gap between science and practice through community engagement and social action to increase health equity. The goal is to make research more responsive to existing needs and to enhance a community’s ability to address important health issues. Thus, successful implementation of the CBPR process is contingent on a collaborative process between community-based organizations and academic investigators [10].

The CBPR approach has been successfully utilized in health disparities research collaborations with disenfranchised small face-to face communities who share common health disparities [11]. This approach is appealing to communities because it prioritizes community-cultural engagement, inter-personal relationships, and includes the community voice throughout the entire research process.

The development of research partnerships between academic institutions and members of communities facilitates active engagement in a mutual process which is used to address an issue of concern of the community group. Typically the selected issue represents a health disparity experienced by the community group. The goal of the CBPR process is to empower the community group to develop their own capacity for developing a realistic and achievable solution to the issue. Solutions that are generated by the community group have the group’s buy-in, and are thus more sustainable. One of the roles of the academic researcher is to provide the community group with knowledge generated from research findings. The community group then translates the knowledge into actionable steps which the group then implements and later on, uses their own results to form their own health policies.

The community group is known as the ‘community partner’ in the CBPR methodology; the academic researchers are known as the ‘academic partners’. The success of the CBPR approach results from equitably partnering academic researchers and community partners. The expertise of the academic partner informs the research design. Since the community partners are those directly affected by the health disparity, they are knowledgeable of the local circumstances that impact health and the feasibility of intervention approaches [12,13]. The success of the community-based research project is determined by the collaborative investment in team building, sharing resources, and mutually exchanging ideas and expertise [14,15,16].

More recently, the literature has begun to address contributions of CBPR to the development of intervention research [17]. Health equity is best achieved when academic researchers form collaborative partnerships with communities. CBPR expands the potential for the translational sciences to develop, implement, and disseminate effective interventions across diverse communities through strategies to redress power imbalances; facilitate mutual benefit among community and academic partners; and promote reciprocal knowledge translation, incorporating community theories into the research [18].

Two potential facilitators to CBPR participation are suggested in the literature. The first is the attributes of the participants: ability to collaborate [19], and capacity for reflection [20]. The second are the situational processes: collaborative responses to challenges [21], group process and conflict techniques [22], and sharing of power and influence [23].

The existing literature tends to focus on either descriptions of researchers’ perspectives in working with academic-community collaborations or the spectrum of strategies used by researchers to engage community members [e.g., 24, 25, 26, 27, 28]. The present study contributes to the literature by (1) assessing the perspectives of community participants about engaging in CBPR and (2) developing a model to guide academic-community partners’ approach in their community interface. The model is developed from our assessments of how community partners perceive what works, what doesn’t, and the relationship between their involvement and nature of the research.

## MATERIALS & METHOD

### The Study Investigators

The Hui A'o Ikaika research team is composed of a senior faculty member representing a large school of nursing at a flagship university (academic partner) and three community collaborators (community partners): the projects coordinator and programs director of a Native Hawaiian health services organization, and a social services director of a locally based human services organization The literal translation (*hui = group, a'o ikaika = discipline*) has symbolized the group’s transformational endeavors as an academic-community partnership. In the process of providing community-based training to research partners [29], we realized that while many resources existed about *how* to engage in CBPR, yet there were few resources about the community’s expectations of approaches of data collectors within their neighborhoods. This led to the development of our study [30].

### Procedures and Data Collection

#### Study Advisory Board

We used a series of qualitative methods to address the research question. Prior to designing the interview guide, we developed a community advisory panel which consisted of ten native Hawaiian elders. The advisory group represented grass roots organizations and individuals with experience in community-based research methods. The advisory board provided oversight of the study, assisted with developing the tools for the key informant interviews and focus groups, identifying and recruiting participants. Informant interview questions were finalized and piloted with the advisory group. The interview guide represented expectations of community members about engaging in participatory research (Table 1).

#### Participant Identification and Recruitment

A diverse group of native Hawaiian community-dwelling participants was purposively sought. Individuals who self-identified as espousing native Hawaiian cultural lifestyle and viewpoints were eligible to participate; it was not necessary for the individuals to meet the contemporary legal definition of “native Hawaiian”, which is a “descendant with at least one-half blood quantum of individuals inhabiting the Hawaiian Islands prior to 1778.”

In the State of Hawai‘i, five Native Hawaiian Health Care Systems exist. This research was performed under the auspices of Ke Ola Mamo, which is the largest native Hawaiian Health Care System in the State of Hawai‘i and encompasses the Native Hawaiian Community on the island of O`ahu. Since the Health Care System covers the entire island, the four local native Hawaiian communities representing the four service districts were included. The districts also provided representation of differences between rural vs urban locations, socioeconomic status and participants’ experience in CBPR methodology. The project was determined exempt by review of the Institutional Review Boards of the University and the Native Hawaiian community.

#### Protocol Development and Data Collection

The staff (N=8) of the community partner organization received community-based focus group implementation training by the academic partner. The community outreach staff, who are trained assistants representing native Hawaiian communities, were the core audience of the training. While only three outreach staff were ultimately engaged in conducting the project’s focus groups, one of the tenets of the CBPR approach is to engage community partner organizations in the entirely of the process. The purpose of this transparency aims to benefit the community-based organization in terms of development of academic-community partner camaraderie, trust development of the research process, and knowledge about use of community participation as an assessment methodology.

The outreach staff engaged in network or snowballing sampling techniques within the four service regions for the focus group recruitment. Data collection consisted of digitally recorded open-ended and semi-structured focus group interviews, addressing community members’ perceptions of CBPR. The focus groups were informally scheduled, based on the convenience of the individuals in that community local. For example, the outreach staff interviewers spontaneously convened a focus group if a minimum of 3–5 interested individuals were present. This strategy was used to increase the convenience of community members’ participation. Twenty focus groups ranging in duration from 30 to 60 minutes were accomplished. The Hui A'o Ikaika research team provided procedural oversight, methodological and technical support for from behind the scenes and debriefing for the three outreach staff interviewers.

This process developed several outcomes consistent with the CBPR methodology: (1) power sharing between the Hui A'o Ikaika research team and the outreach staff interviewers who represented several different communities; (2) trust facilitation from the research team to the interviewers, and from the outreach staff interviewers to the participant members representing local communities; and (3) mentored research skill development by the academic partner.

### Data Analysis

The Hui A'o Ikaika research team was responsible for the analysis. Each focus group interview was transcribed verbatim. The qualitative data analysis software NVivo version 10 assisted with data management. The process of inductive content analysis involving a constant comparative approach with no a priori coding was used. The major goal of this stage was to immerse in the data and gain a sense of the major themes.

Descriptive statements were formed and an analysis was carried out on the data under the questioning route. First, each member of the research team read the transcripts and generated initial coding categories. The research team discussed the coding categories, developing a schema and finalizing the final coding categories through consensus. Subsequently, the research team members formed two pairs and each pair independently coded each interview. The entire group of four researchers then reassembled to cross check the interview coding and discuss any coding discrepancies.

Indexing followed, which included highlighting and sorting out quotes and making comparisons both within and between participants/groups. The next step involved rearranging the quotes under the newly developed themes. Discussions continued until consensus was reached. Data saturation was noted during the final iterative reviews of the themes. The text analysis was then reviewed by an independent coder who also provided secondary verification of data saturation. The team then engaged in the final step of interpreting the linkages and relationships within the entirety of the data. The team discussions led to the development of a model depicting community members’ perceptions of community-based research.

Respondents indicated a gradient of how much to invest in community-based research participation. There was a general agreement that individuals had a choice about participating. Participants pointed out their power to not participate and cause the project’s demise or in contrast, to contribute some type of expertise to enhance the project.

“Be a team player and share information, or not be a team player. We are our own experts because this research is situated in our community. Everyone doing what they’re good at if we like it [the research idea].”

“Participation in different roles possible, advocate, educate, mentor, planner … I decide if I take leadership role in all aspects of research from start to finish.”

#### Theme 2. Perceptions about the purpose, research intent and expectations led to the vetting of researchers

Respondents identified key beliefs that research teams should match the community’s culture, norms and mores. Patience is needed to determine the types of researchers who can blend with the community. Routine and casual interactions were identified as important in the researcher selection process. The participants expressed it was the responsibility of the research team to adapt themselves to the culture in the community. The main viewpoint may be summarized as the fit of the research agenda with the community’s realities. Four supporting key viewpoints guided how communities elected to partner with research teams.

- Personal relationships were conveyed as key to establishing the credibility of the research team. These included personal connections, word of mouth, as well as past knowledge and experience. Specific individuals were identified to meet the community’s needs.
- The local media was depicted as a credible source for obtaining assistance in research engagement. Participants conveyed an underlying expectation of the “distance” to which their community problems should be ‘broadcasted” in their quest for seeking assistance. Going too far was seen as “showing the dirty laundry” yet not reaching far enough would not bring the needed resources. For example, use of the local media provided the vehicle for fostering interaction among communities with neighboring communities, while broadcasting the issue throughout a city may be deemed too broad in scope. Participants described using the local media to create, share or exchange information and ideas in among networks. Several social media options were named, such as Face Book, local radio stations and the local TV station “`Ōlelo”. One active community developed a network called “Red Alert”, consisting of simultaneous use of several modalities, such as email blasts, posting on bulletin boards, and word-of-mouth to keep their community members informed.
- Existing local organizations and groups were identified. Institutional factors perceived to aid communities were ones that made use of existing community structures, provided an array of needed services, and had knowledge of how the local system worked. Participants described establishing trustable linkages in a “step ladder approach” – that is, building relationships one step-at-a time. The view held by the group was that the community trusts its own members, and hence, the agency if some of their own were employed at the site.
- Finally, community gatherings were identified as mechanisms to garner and locate resources for addressing the community issues. The benefits of this approach enabled participants to use all three modalities: knowledge of personal relationships, evaluation of local media, and sorting through local organizations based on others’ experiences.

#### Theme 3. Expectations of roles and responsibilities of the researcher(s) reflected meaningfulness of the interaction

Many participants assigned attributions to the research team which then influenced the depth of the CBPR partnership. The research team is expected to balance their participation with other roles and responsibilities. Comments focused on the team’s performance at multiple levels of investment. The levels of community investment were described in terms of strength, ranging from weak to strong.

Validity of the findings was enhanced by the involvement of volunteers solicited from each focus group to participate in validation of the data categories and themes developed by the research team. In accordance with traditional qualitative methodologies [31], we initially planned to meet in person with the volunteers to confirm or challenge the accuracy of the work. However the volunteers indicated it was more convenient to receive the materials for review electronically (e-mailed attachments), or via phone follow-up to mailed documents.

## RESULTS AND DISCUSSION

Although all participants indicated identification with native Hawaiian culture, sixty-three percent of the sample indicated native Hawaiian ancestry. Slightly over half (fifty-seven percent) reported having no experience in CBPR. Those with CBPR involvement had slightly over two years’ experience and self-rated their level of CBPR experience as moderate (6.7 on a scale of “1” (low) to “10” (high). (Table 2). The roles reported by participants indicating prior experience with CBPR are shown in Table 3.

Three key areas were identified: (1) reciprocal trustable is needed; (2) perceptions about the purpose, research intent and expectations; (3) expectations of roles and responsibilities of the researcher(s). (Table 4).

### Theme 1. Reciprocal trust is needed between the academic researcher and the community partners

In order to develop trust, participants identified expectations of the researchers as they approached the community. The notion of “circles of relationships” evolved. The “circles” represented different boundaries in relationships between researchers and community participants. For example, prior to entering into a collaborative approach, a boundary perceived by community members was the need to develop respect for the needs of the community first before introducing what the research team would lend to the process.

The notion of the “circle of relationship” was also depicted as the research team’s role to bring [new] information from outside the community’s “circle”, e.g., news of related events from other communities. Knowing the experiences of other communities provides a springboard for negotiations with the research team. These types of negotiations represent boundary settings. There was an expectation that researchers must be flexible to changes in the relation boundaries that occur as the research project evolves.

“Hear what we need to provide support to us. It depends. It can change. Some groups want to learn how to get own resources, and maybe others want to learn skills [capacity], access to resources.”

An expectation was the researchers had responsibility to take the lead to build relationships with the community. The research team was seen as the entity entering the neighborhood domain.

“Make it so both parties can learn from each other, talk community language “local jargon”, use a “win-win” approach, how do we trust you, recognize our leaders, tell us how to get involved, spend a lot of time to get to know us, speak up [advocate] for our community issues and join our voice.”

There was general agreement that the context/activities of research activities gave meaning for research. The topics, even if proposed by the academic partners, will determine the amount of energy to be invested. There are many perspectives of the worthiness of a topic.

“[The] Research must be helpful to the community, help the community to discover something they didn’t realize, finding resources to make the community better, recognize that every community is unique, involve from start through all the stages.”

“Participation is linked with the purpose [of the study] and [our] expectations. Our community must be clear about our issues [problem], topic needs to be meaningful. We need follow-up on past results.”

“As a researcher, you need to work on keeping the intervention going and not stop it after the grant or it will not benefit the future, such as our kids. Sustainability means leaving the equipment so we can still use it when you are done.”

Five areas were identified that include the research team’s prior knowledge of the community, their actions and appearance, gaining community support, and the researchers’ characteristics.

Participants identified the importance of knowing the community before coming into it. Respondents indicated a strong sense that each community is different. The research team’s experiences with one group may serve as a guide for their approach to another group, but there is always a need to address the unique differences.

“Know how our community came to be, be familiar with our history and lifestyle.”

“Learn values and priorities of our community, respect people’s time [by learning beforehand], and talk on our level.”

“This includes appropriate greetings and proper dress [of the researcher] in different situations.”

“Who controls the power changes depends on where you are.”

Research teams’ actions before, during and after the interactions in the community were evaluated by the participants. Participants’ expectations were that research teams use approaches to foster community collaborations. The research team was seen as given responsibilities in exchange of entering the community and collecting data. These responsibilities focused on professional, responsible and ethical conduct of research.

“Select projects that make sense to us – ask us. Don’t guess, don’t be biased.”

“They should start small and get it right before making the project large.”

“What is the follow through? Can they keep us informed, use our ideas and don’t just collect it? Can they help us come up with the benefits, and help us solve our problems?”

“You should share your project’s resources if you want us to join you.”

“I’m concerned if you have a pre-set agenda. You will not represent our concerns correctly.”

There was high agreement of the need to gain community support. For example, gaining support included making sure the research is needed by the community, entering into the community, and being open to the specific situation-at-hand.

“Can they make sure the results are valued by the community? We are proud of our community.”

“Ask community permission, find the right leaders, find community members who can vouch for you before you come into our neighborhood.”

“Put yourself out there and meet us, but don’t be intrusive. If you come to recruit, know how far to go – [for example] you cannot come up my steps and bang on my door at dinner time [or I will feel like I have to ask you in and feed you dinner].”

“Don’t compare [pre-judge] us with those other groups.”

The researcher characteristics were also noted as either facilitating or posing a barrier when interfacing with the respondents. There is an intricate network within each community. As researchers enter the community, a process of decoding community expectations is needed.

“Be respectful, be accessible, willing to accept what is said. Have honesty about your project, open, understand how to read between the lines, go with the flow, i.e. expect the unexpected, humble and accept mistakes.”

“Watch how you come up. Don’t come up so close to my house. Know your distance or if I don’t like you, my dog will bite you.”

### Model Development

**Figure 1:**
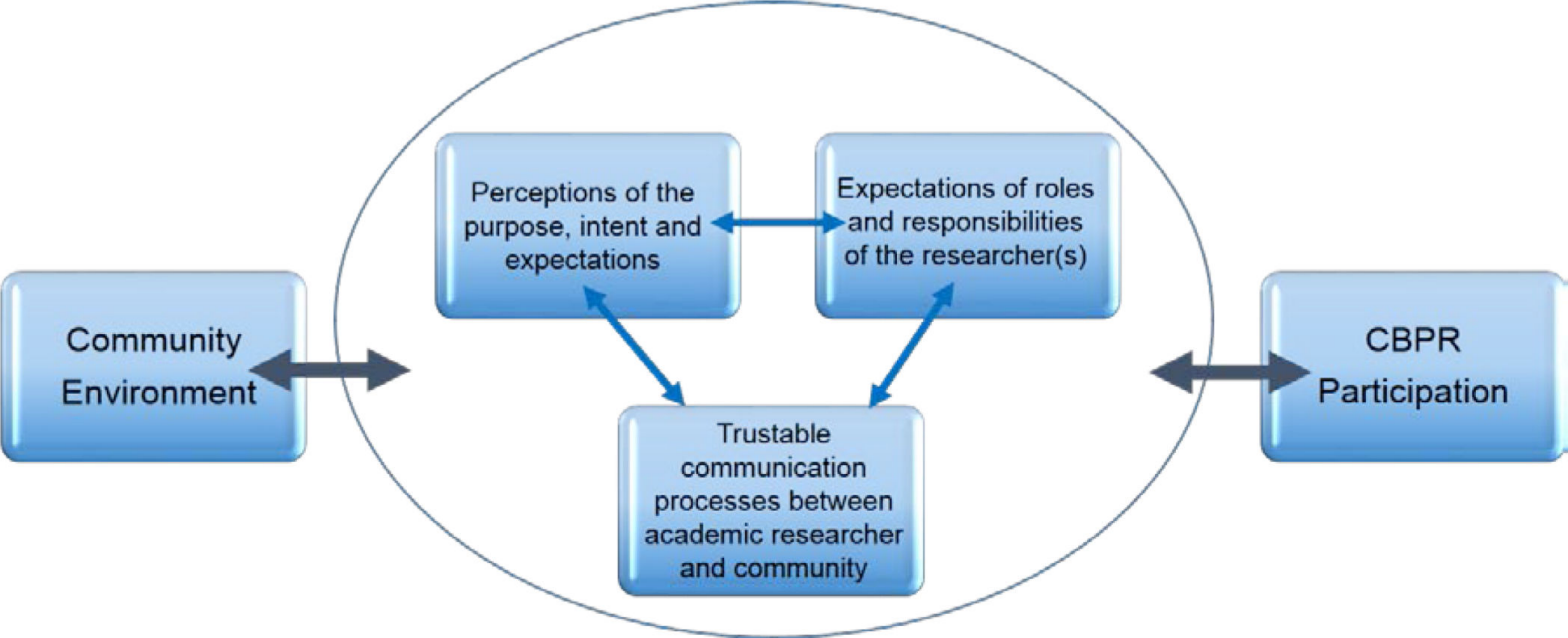
Model of community members’ perceptions of community-based research

The three key themes described the community’s relationships and expectations of academic partners. By reviewing the context and repetitions in the data, we noticed that the three themes implied reciprocal relationships. This resulted in three dimensions influencing community members’ perceptions of community-based research. (Figure 1)

According to Figure 1, community participants perceived they have a responsibility to be the gateway for a research team to engage with them. Even CBPR teams founded within the community require approval for access by the individuals whose data will be collected. The suggestion was made to develop trustable linkages in a “step ladder approach”, building relationships one step-at-a time. The identification of acceptable researchers and research teams then leads to the delineation of levels of trustable communication and the level to which participants will engage in research activities. As the communication and activities develop, the group establishes a relationship between what they are perceiving and their expectations of the researcher/research team’s roles and responsibilities. These factors determine the level to which the community group is willing to engage.

### Discussion

The concept of “readiness of an academic-community partnership” is described by Andrews [32] as (1) goodness of fit; (2) capacity; and (3) operations. Expectations of the community and academic partners influence the readiness of partnerships. Andrews depicts CBPR partnership readiness in a model that begins with antecedents (mutual interest) which influences a triadic relationship consisting of (1) the goodness of fit (shared values, compatible climate and mutual beneficial commitment); (2) capacity (effective leadership, inclusive membership complementary competencies and adequate resources); and (3) operations (congruent goals, transparent communication, conflict resolution and equal power). Intermediate outcomes (sustainable partnership/product, mutual growth and policy) as well as long term outcomes (social and health impact on community) are either achieved or not achieved; however, this relationship is not static.

**Figure 2:**
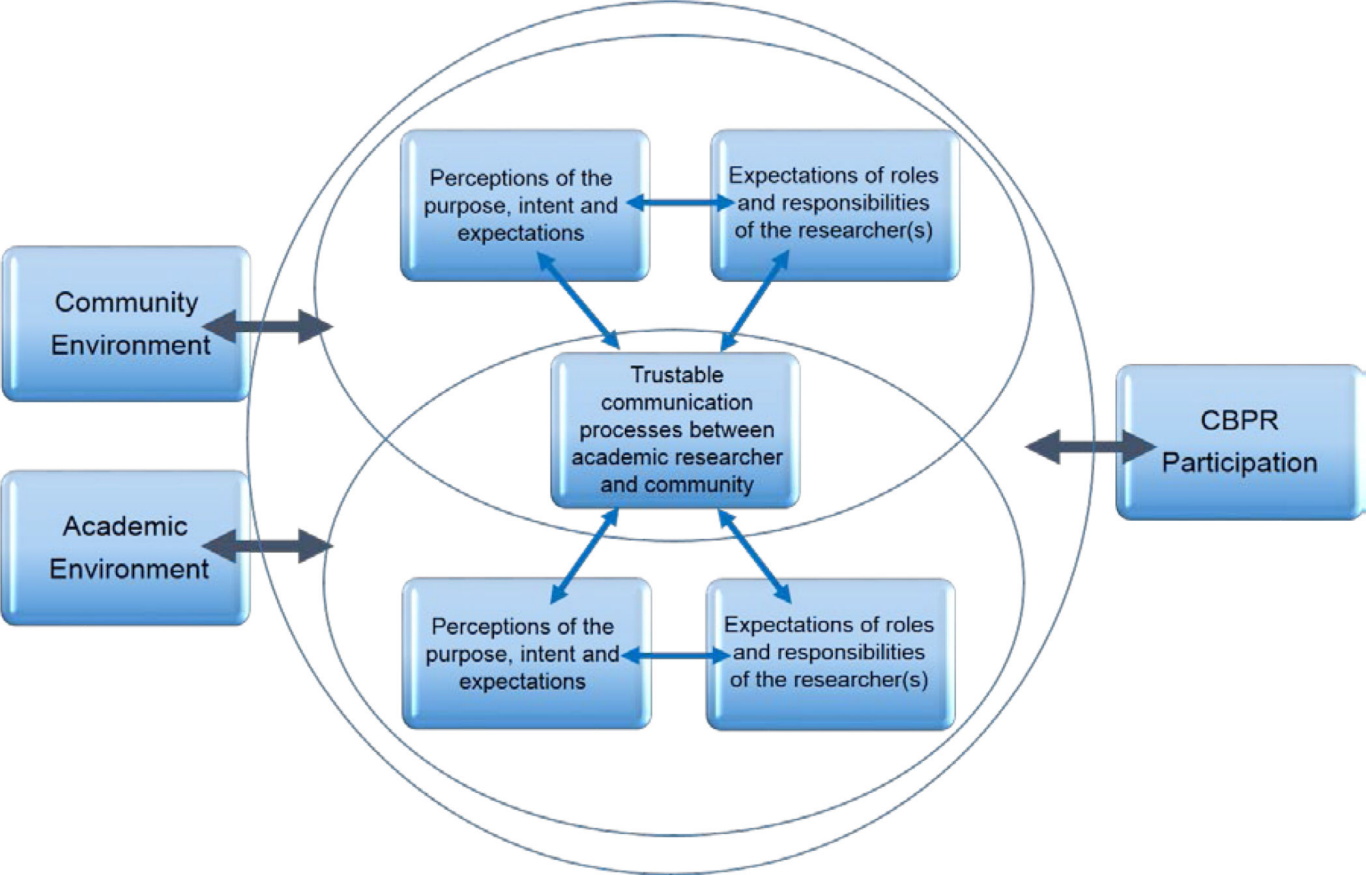
Proposed model of engagement in CBPR partnerships

Our findings support Andrew’s CBPR Partnership Readiness Model. CBPR participation is an iterative and dynamic process, partnership and issue specific, influenced by a range of environmental and contextual factors, amenable to change and essential for sustainability and promotion of health and social change in the community. As all academic-community based research activities occurs in partnerships, the relationships depicted by the three key themes in Figure 1 requires a similar concurrent process to occur among the academic research partners. The reciprocity between the academic partner and the community partner is needed to establish the full CBPR process. Thus we propose the Model shown in Figure 2, which includes the dynamics between the agencies of the community partners and academic partners.

According to Figure 2, the interaction with academic partners is integral to the CBPR process. Academic researchers must have a strong orientation toward collaborating with community partners. Trustable communication and activities are inter-group attributes that form the key bridge between community and academic partners [33]. Likewise, academic partners hold expectations of roles and responsibilities of community partners [34,35,36].

Since the interactions shown in Figure 2 between the academic and community partners is hypothesized, we recommend adapting and using the interview guide with academic partners who address health issues within the same geographical setting as their community partners. The results may be used to confirm the proposed relationships in Figure 2.

There were four limitations in this study. First, the results represent the opinions of the participants who agreed to participate in a geographical area of one state. Second, the timing of the interviews engaged more female participants than males. Third, although we sought geographical representation, only participants from four communities were sampled. Lastly, the majority of the participants were native Hawaiian or reported affiliation with the native Hawaiian culture and their expectations may differ from other populations.

## CONCLUSIONS

The purpose of this focus group study was to describe community’s’ perceptions of engaging in CBPR. Much has been written about involving community members and the implementation of the CBPR process, yet limited information exists about expectations of community partners who actually engage in the process. The results of this study indicate that trustable communication processes are needed between the academic researcher and the community, there is a need for locating researchers that fit the community, and delineation of expectations about the roles and responsibilities of the researcher(s) is important. These types of expectations would inform the types of training that would best facilitate both academic and community partner’s participation in CBPR. The success of the community-based participatory research methodology to address challenges of translating research findings into community actions is enhanced when the expectations of community members are taken into account.

## Figures and Tables

**Table 1:** Key informant interview questions

What does community research mean to you? What roles do people typically take on when they participate in community research? What are your feelings about research being done in communities? What are the benefits? What are the drawbacks? Why would communities want to get involved in doing research? What would make communities want to participate in community research? What would make communities not want to participate in community research?

**Table 2:** Characteristics of focus group participants (N=56)

	Female (N=43)	Male (N=13)	Total Percent
Racial categories (self-reported)			
American Indian/Alaska Native	0	0	
Asian	7	0	12.5%
Native Hawaiian	28	7	62.5%
White	3	1	7.1%
More than one Race	1	4	8.9%
Unknown or Not Reported	4	1	8.9%
Self-reported prior involvement in CBPR			
No	24	8	57.1%
Yes	19	5	42.9%
Years of CBPR experience (mean)	2.1 yrs.		
Self-rated level of CBPR experience [1=low; 10=high] (mean)	6.7		
Educational Level (mode)	High school diploma/GED or some college/ technical		
Age (mean)	44.9 yrs.		

**Table 3:** Previous roles in community research projects (n=10)

Answered questions of multiple questions
Assistant on a few different CBPR projects at a community-based cancer services agency
Data collection
Educator, advocate
Identifying community needs. Applying completed research to practice
Participant and advocate for the Native Hawaiian Health Systems
Principal investigator, analyst/evaluator depending on project
Research assistant
Surveyor and clerk
Visit to town [for a focus group] and got paid

**Table 4:** Themes and Subthemes

Theme	Subthemes
Reciprocal trust is needed between the academic researcher and the community partners	Circles of relationships representing levels of boundaries Researchers have to build relationships with the community Context/activities of research activities gives meaning for research Participation is linked to the purpose and expectations
Perceptions about the purpose, research intent and expectations led to the vetting of researchers	Personal relationships Local gatherings Local media Relationships with local organizations and groups
Expectations of roles and responsibilities of the researcher(s) reflected meaningfulness of the interaction	Know the community before coming into it Researcher’s actions and appearance Gain community support Researcher characteristics
